# TRPM8 Ion Channels Differentially Modulate Proliferation and Cell Cycle Distribution of Normal and Cancer Prostate Cells

**DOI:** 10.1371/journal.pone.0051825

**Published:** 2012-12-14

**Authors:** María Ll. Valero, Fernanda Mello de Queiroz, Walter Stühmer, Félix Viana, Luis A. Pardo

**Affiliations:** 1 Department of Molecular Biology of Neuronal Signals, Max-Planck Institute of Experimental Medicine, Göttingen, Germany; 2 Instituto de Neurociencias de Alicante, Universidad Miguel Hernández-CSIC, Alicante, Spain; 3 DFG Research Center for Molecular Physiology of the Brain (CMPB), Göttingen, Germany; Albany Medical College, United States of America

## Abstract

Overexpression of the cation-permeable channel TRPM8 in prostate cancers might represent a novel opportunity for their treatment. Inhibitors of TRPM8 reduce the growth of prostate cancer cells. We have used two recently described and highly specific blockers, AMTB and JNJ41876666, and RNAi to determine the relevance of TRPM8 expression in the proliferation of non-tumor and tumor cells. Inhibition of the expression or function of the channel reduces proliferation rates and proliferative fraction in all tumor cells tested, but not of non-tumor prostate cells. We observed no consistent acceleration of growth after stimulation of the channel with menthol or icilin, indicating that basal TRPM8 expression is enough to sustain growth of prostate cancer cells.

## Introduction

TRPM8 is a calcium-permeable, non-selective cation channel of the transient receptor potential superfamily [1], required for the transduction of moderate cold temperatures [2], [3]. The presence of TRPM8 in cold-responsive small-diameter neurons in dorsal root ganglia and trigeminal ganglia and the phenotype detected in TRPM8−/− knockout mice supports a role of TRPM8 in thermosensation and nociception [4]–[6]. TRPM8 channels have been cloned from species in different genera, from amphibians to humans [7]. Human TRPM8 was initially identified during a screen for up-regulated genes in prostate cancer (and therefore termed trp-p8 [8] but later detected in other tumor types [9], [10]. Among normal tissues the expression of the channel is very restricted to a subpopulation of primary sensory neurons [2], [3], but it is also present in the male reproductive system in significant amounts [2], [3], [8], [9], [11], [12].

Activation of endogenous (i.e. neuronal) or recombinant TRPM8 channels gives rise to a signature current characterized by extreme outward rectification and voltage-dependent gating [13]–[15]. TRPM8 channels can be activated by specific and selective agonists, either natural (such as eucalyptol and menthol) or synthetic compounds like the super cooling agent icilin, which is so far the most potent agonist of TRPM8 [2], [3], [16]–[19]. Other agonists (linalool, geraniol, among others) were identified by screening menthol derivatives or odorant compounds. In particular, geraniol might be a physiological activator of TRPM8 because it is an intermediate during cholesterol synthesis and it induces proliferation in prostate epithelium. All known TRPM8 agonists induce a cooling effect, reinforcing the concept of a role of TRPM8 in cold perception [20].

TRPM8 mRNA has been detected in malignant cells, and this has been extensively studied in prostate cancer. TRPM8 mRNA was highly overexpressed in well-differentiated early prostate tumors. In a typical model for androgen-dependent prostate cancer (LNCaP cells; epithelial apical cells with a secretory phenotype) expression is detected at both the plasma membrane and the endoplasmic reticulum, where it could act as a Ca^2+^ release channel [18], [19], [21]–[23]. Plasma membrane TRPM8 might exert a protective effect, since activation of TRPM8 by PSA (prostate specific antigen) reduced cell motility in PC3 cells [24].

TRPM8 might be a useful marker for prostate cancer outcome, since loss of TRPM8 expression appears to be associated to transition to androgen independence and poor prognosis [19], [21], [25]. This might reflect the effect of androgens on TRPM8 expression, since the gene displays ten putative androgen responsive elements [18]. Abnormal levels of TRPM8 mRNA can also be indicative of metastatic disease [26].

Canonical TRPM8 channel function can be blocked by urea compounds (see below), which are also known to inhibit TRPV1 [17], [25], [27]. This limits the use of such blockers in the study of the role of TRPM8 in prostate cancer because the cells express also TRPV1 [28]. At present, the only feasible way to specifically dissect the role of the channel in prostate cancer is the use of siRNA. RNA interference can produce an effective and specific knock down of a particular gene *in vivo* and *in vitro* [29], [30].

In this study we examined the effects that TRPM8 silencing has on cell survival and cell proliferation of three human prostate cells lines, LNCaP, PC3 and DU145. LNCaP is strongly androgen-dependent [19] while DU145 is androgen-independent [31] and PC3 would represent an intermediate status [32]. We also studied the behavior in this respect of a non-tumoral (SV40 immortalized) prostate cell line, PNT1A [33]. Inhibition of the expression or function of the channel reduced proliferation rates and proliferative fraction in all tumor cells tested, but not of non-tumor prostate cells.

## Materials and Methods

### Cell Culture

Prostate-derived cell lines PNT1A (ECACC 95012614), LNCaP (DSMZ ACC 256), PC3 (DSMZ ACC 465) and DU145 (DSMZ ACC 261) were obtained from DSMZ (Braunschweig, Germany) or ECACC (Salisbury, UK). Each line was propagated and maintained according to the instructions of the corresponding provider. Identity of tumor cell lines was confirmed through expression of specific markers (**PC3** AR−, ERα+, ERβ+, PSA−, DD3−; **DU145** AR−, ERα−, ERβ+, PSA−, DD3−; **LNCaP** AR+, ERα−, ERβ+, PSA+, DD3+; Paul Thelen, Department of Urology, University Hospital Göttingen).

### siRNA Transfections

Transfection with siRNAs (25 nM) was performed using either Lipofectamine 2000 or Lipofectamine RNAiMAX reagents in OptiMEM medium (Invitrogen GmbH, Karlsruhe, Germany) 24 hours after plating. Four different siRNAs were designed to target hTRPM8 channel using the HiPerformance siRNA Design Algorithm (Qiagen). The following hTRPM8 (NM_024080) sequences were used: TRPM8.1, tcgaatgttctcacctattaa; TRPM8.2, aaggttagattccaataaata; TRPM8.3, cagaatgttatcatactacat and TRPM8.4, ccgggacgagatggacataga.

All siRNAs were synthesized by Qiagen (Hilden, Germany), except the commercial Negative Control #1 and the human GAPDH siRNA (Ambion, Darmstadt, Germany), which we used as negative and positive controls, respectively. The cells were incubated with the siRNA and the transfection reagent for 6 h and harvested for the experiments just after the end of transfection. Additionally, cells treated only with OptiMEM and the corresponding transfection reagent were included as controls.

### qPCR

Total RNA obtained from cultures using RNeasy mini kit (Qiagen, Hilden, Germany) was reverse transcribed (SuperScript, Invitrogen, Karlsruhe, Germany) with oligo-dT and gene specific primers for hTRPM8 (5′-cttgggcaaaacacacaatg-3′). Real-time PCR was performed on the template using the TaqMan system in an AbiPrism 7700 Sequence Detector (Applied Biosystems, Foster City, CA) or a LightCycler 480 (Roche, Mannheim, Germany). The following fragments were amplified: nt 2910–3058 from sequence NM_024080.4 was detected with the hTRPM8 probe (5′-FAM-tcgccatgtttggctacacggtgggcacc-TAMRA-3′) and nt 1632–1732 from sequence NM_003234 was detected with the hTFR probe (5′-JOE-tgaatggctagagggatacctttcgtccc-(6-TAMRA)-3′). The human transferrin receptor was used as a control template for RNA integrity and PCR performance. Relative quantification was performed using the REST software (Relative Expression Software Tool; [34], [35]).

### Flow Cytometry

siRNA: After treatment with siRNA, cells were plated in 6-well plates and were incubated for 24–120 h before measurements. Cells were trypsinized, washed twice with PBS and subsequently kept on ice. For cell cycle measurements, the cells were incubated with 50 µg/ml propidium iodide (PI), 0.3% saponine and 100 U/ml RNase. For viability measurements, non-viable cells were detected by staining with 10 µg/ml PI in PBS. Samples were analyzed on a BD FACSAria flow cytometer (Becton Dickinson, Heidelberg, Germany). Propidium iodide was excited at 488 nm, and fluorescence was collected using a 595 nm dichroic mirror and 610/20 nm band-pass filter (for cell cycle) or 655 nm dichroic mirror and 695/40 nm band-pass filter (for viability). Gates for viable cells were established by forward- and side scatter and cell cycle phases were determined using the FlowJo software (Tree Star Inc., Ashland, USA). Alternatively, the percentage of viable cells was directly determined by PI exclusion.

For experiments in the presence of menthol and icilin, cells were plated in 6-well plates and incubated for 24–168 h with 125–300 µM menthol or 10 µM icilin before measurements. Cells were trypsinized, and washed twice with PBS. Samples were treated and analyzed for cell cycle and viability measurements as described before.

### Calcium Imaging

The calcium imaging experiments were conducted with the fluorescent indicator Fura-2. Prior to each experiment, the cells were incubated with 5 µM acetoxymethylester form of Fura-2 (Fura-2AM; Invitrogen) for 45 min at 37°C. After this, 250 µM of sulfinpyrazone was added for another 20 min in order to block the anion transporter that impairs the loading with Fura-2AM in the cancer cells lines. Fluorescence measurements were made in a Leica (Nussloch, Germany) DM IRE2 inverted microscope equipped with a 12-bit cooled CCD camera (Imago QE Sensicam; TILL Photonics, Graefelfing Germany). Fura2 was excited at 340 and 380 nm with a Polychrome IV monochromator (TILL Photonics) and the emitted fluorescence was filtered through a 510 nm long-pass filter. Calibrated ratios were displayed on line at 0.5 Hz using TILL Vision software version 4.01 (TILL Photonics). The calcium imaging experiments were performed simultaneously with temperature recordings. The bath solution, referred to as ‘control solution’, contained (mM): NaCl 140, KCl 3, CaCl_2_ 2.4, MgCl_2_ 1.3, Hepes 10 and glucose 10, and was adjusted to pH 7.4 with NaOH.

### Proliferation Assays

Proliferation was estimated based on the ability of metabolically active cells to reduce tetrazolium salts to colored formazan (3-(4,5-dimethylthiazol-2-yl)-2,5-diphenyltetrazolium bromide, MTT; Sigma-Aldrich). Cells were trypsinized and plated in flat bottom 96-well plates at densities ranging from 2000 to 5000 cells/well depending on the cell line examined. To determine the metabolic activity, 10 µl of MTT reagent were added to each well and incubated for 4 h. The absorbance of the produced formazan was determined in a Victor2 plate reader (Wallac) using 570 nm and 630 nm excitation filters. For experiments with menthol, the medium was renewed every 48 h both in the test and control wells.

### Wound Healing Assay

Cells were first cultured to confluence (>90%) in 6-well dishes. A small area was then disrupted by scratching the monolayer with a 1000 µl plastic pipette tip. Thereafter, the cells were cultured for 12, 24 or 48 h under different experimental conditions: low serum or either vehicle or drug-containing medium. Cells were inspected microscopically over time. The remaining wound area was calculated using CorelDraw software and the migration distance of the cells was estimated based on that calculation.

### Drugs

**Figure 1:**
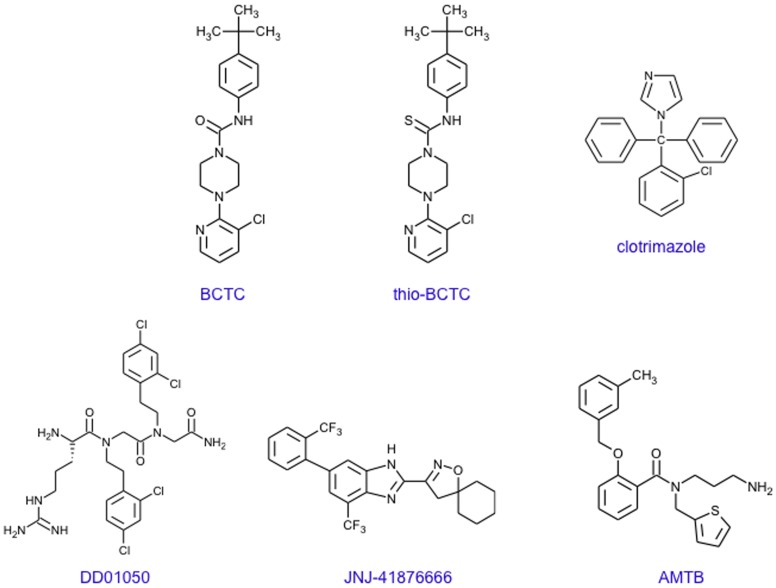
Chemical structure of the drugs with TRPM8 antagonist activity used in this study.

4-(3-Chloro-pyridin-2-yl)-piperazine-1-carboxylic acid (4-tertbutyl-phenyl)-amide (BCTC) was a generous gift from Grünenthal AG (Aachen, Germany). [L-arginyl]-[N-[2,4-dichlorophenethyl]glycyl]-N-(2,4-dichlorophenethyl)glycinamide (DD01050; (H-Arg-15-15 C in [36]), was a gift from Dr. A. Ferrer-Montiel (Universidad Miguel Hernández, Spain). AMTB (N-(3-aminopropyl)-2-[(3-methylphenyl)methoxy]-N-(2-thienylmethyl)-benzamide hydrochloride (1∶1) hyclate) a novel, highly selective TRPM8 antagonist was a generous gift of Dr. Stuart Bevan (King’s College, London). JNJ41876666 (compound 5 in reference [37]; 3-[7-Trifluoromethyl-5-(2-trifluoromethyl-phenyl)-1*H-*benzimidazol-2-yl]-1-oxa-2-aza-spiro[4.5]dec-2-ene Hydrochloride,), a potent TRPM8 antagonist, was a generous gift of Janssen Research & Development, LLC (Spring House, PA). Fig. 1 shows the chemical structures of the drugs used.

### Statistical Analysis

Data are presented as mean ± S.E.M. obtained from at least three independent experiments. Statistical significance was evaluated by Student’s t test. P values are indicated in the figures by asterisks near the corresponding column or symbol. * p<0.05; ** p<0.01; *** p<0.005.

## Results

### Detection of TRPM8 Expression in the Prostate and Prostatic Cell Lines

TRPM8 expression is most abundant in nervous tissue and the male reproductive system. Previous studies described the overexpression of TRPM8 in prostate tumors and cell lines derived from prostate cancer, specifically LNCaP [8]. Other cell lines were found negative in those reports. More recently, PC3 cells were reported weakly positive by western blot [21]. We set to determine expression levels of TRPM8 in the prostate cancer cells LNCaP, PC3 and DU145 and the non-tumoral cell line PNT1A by different approaches to expand and complement results reported before [38].

**Figure 2:**
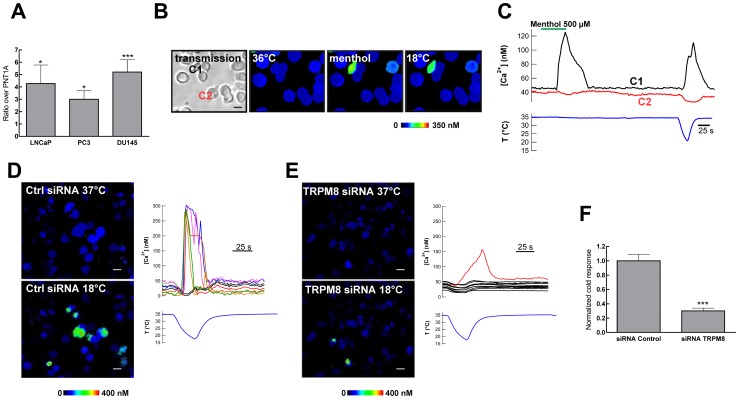
Expression and functional analysis of TRPM8 in prostate cancer cells. **A.** mRNA abundance was determined by real time PCR on cDNA derived from RNA from the indicated source. The human transferrin receptor was used as reference housekeeping gene. RNA abundance is expressed as normalized values over PNT1A. Asterisks indicate statistical significance with respect to PNT1A. **B–C.** DU145 cells respond to cold and menthol**.** **B**. Transmitted (left) and pseudocolor ratiometric [Ca^2+^]_i_ images showing an example of the response of DU145 cells to cold (18°C) and menthol 500 µM. **C.** [Ca^2+^]_i_ responses of a cold- and menthol-sensitive cell (C1) compared to a cold-insensitive, menthol-insensitive DU145 cell (C2). **D–F.** Response to cold is diminished by TRPM8 knockdown in DU145 cells. Pseudocolor ratiometric [Ca^2+^]_i_ images in cells transfected with control siRNA (**D**) or with TRPM8.4 siRNA (**E**) at 37°C (upper panels) or 18°C (lower panels). Individual [Ca^2+^]_i_ responses are represented to the right of the corresponding images. The amplitude and fraction of cells that respond to cold stimuli is clearly diminished in TRPM8 siRNA-treated cultures. This effect is quantitatively depicted in **F.**

First, TRPM8 mRNA content was determined by reverse-transcription real-time PCR (qRT-PCR) using TaqMan probes in normal human brain, prostate, and cell lines LNCaP, PC3, DU145 (derived from tumors) and PNT1A (immortalized non-transformed prostatic cell line). RNA integrity and reverse transcription were controlled by using the human transferrin receptor as reference for normalization. mRNA for TRPM8 was detected in brain and prostate, with maximum levels in the healthy prostate (not shown). The message was also detected in all human cell lines tested, LNCaP, PC3, DU145 and PNT1A. As reported previously, levels in the non-tumoral line PNTA1 were significantly lower than in the cancer cell lines. The relative amount of TRPM8 message was DU145≈LNCaP>PC3>PNT1A (Fig. 2A).

From these results, we conclude that TRPM8 is expressed in all prostatic cell lines tested. Furthermore, the protein appears to form functional channels. In our hands, DU145 (Fig. 2B, C), LNCaP and PC3 (data not shown) cells show an increase in intracellular Ca^2+^ levels upon stimulation by menthol (500 µM) and moderate cold (decrease in temperature from 36°C to 18°C), compatible with the functional expression of TRPM8. Cold-evoked responses in DU145 cells were strongly reduced by siRNA directed against TRPM8 (Fig. 2D–F).

### Effect of TRPM8 Blockers on the Proliferation of Prostate Cancer Cell Lines

The experiments described so far support the notion that TRPM8 expression occurs both in normal prostate and tumor cells. The question remains, however, whether inhibition of TRPM8 has similar effects in all cell types. Our main goal was to test whether TRPM8 activity is a general requirement for the proliferation of different prostate cancer cell lines. To test this, we used pharmacological TRPM8 blockers [39] and siRNA technology.

**Figure 3:**
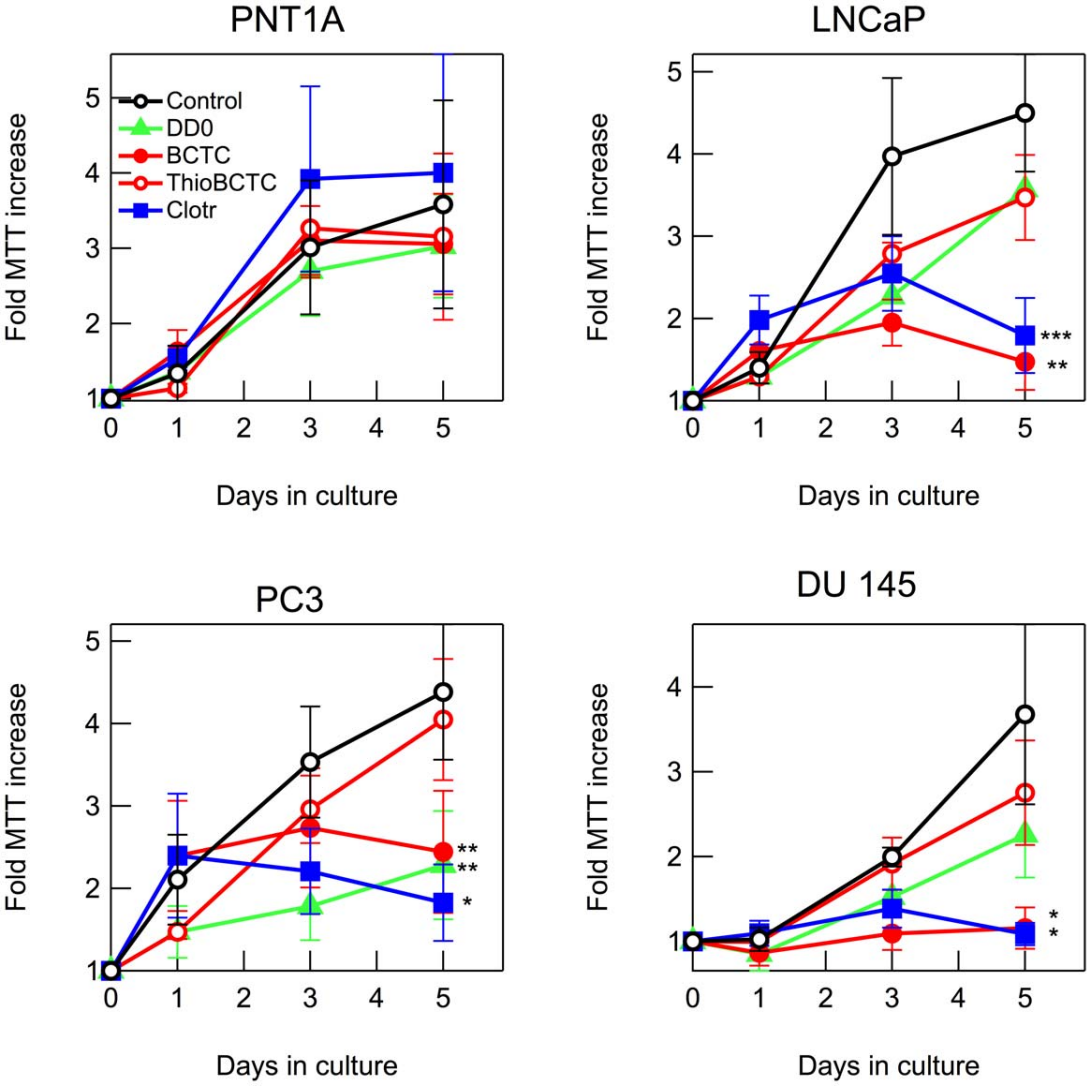
TRPM8 channel blockers reduce proliferation in prostate cancer cells. Growth of the indicated cell lines in the presence of TRPM8 inhibitors. MTT hydrolysis, expressed as fold increase, has been normalized to levels at time 0. Clotrimazole (solid squares), BCTC (solid circles), thio-BCTC (open squares) and DD01050 (triangles) over 5 days of experiment. Data points represent average of 4 experiments ± SEM. *: *p*<0.05; **: *p*<0.01; ***: *p*<0.005. The blockers were used in a final concentration of 10 µM.

**Figure 4:**
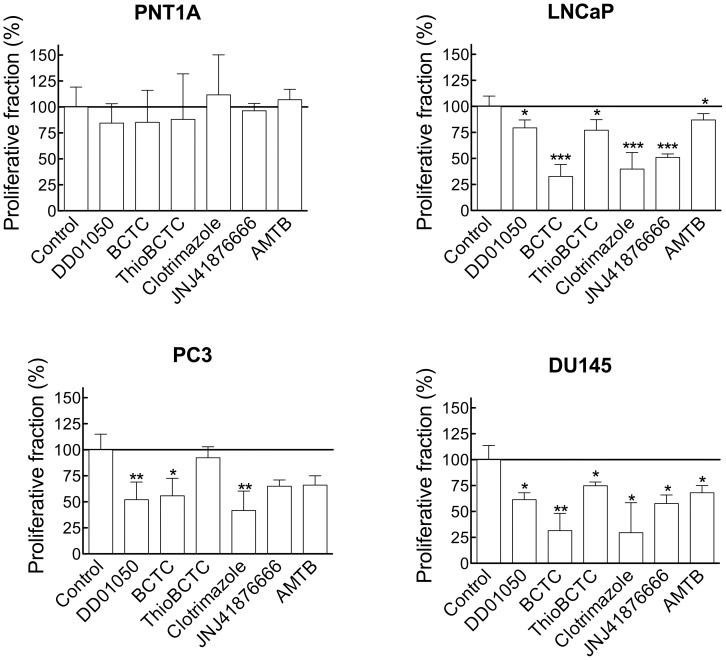
Pharmacological TRPM8 block affects the cell cycle of prostate cancer cells. Histograms show changes in the proliferative fraction in the presence of channel blockers as determined by flow cytometry cell cycle analysis. All drugs were used at a concentration of 10 µM. The proliferative fraction under control conditions (vehicle-treated cells) has been considered as 100% of proliferation.

Blockers used (Fig. 1) were clotrimazole (EC_50_ 200 nM) [40]; BCTC, a blocking agent of TRPV1 channels [41] and of mouse, rat and human TRPM8 channels (with EC_50_ of approximately 0.6–1 µM) [13], [17], [27], [42]; thio-BCTC, a less active (EC_50_ 3.5 µM) analog of BCTC; and DD01050, a novel TRPV1 channel blocker which also blocks TRPM8 in neurons and HEK293 cells (EC_50_ approximately 1 µM) [38]. Proliferation was determined using MTT assays (Fig. 3) and the effect of these drugs and a more selective TRPM8 antagonist (see below) on the cell cycle distribution and cell viability were determined by flow cytometry (Fig. 4).

Clotrimazole is a well-established inhibitor of cancer cell proliferation probably through multiple targets, including the intermediate conductance calcium-activated K^+^ channels [43]. At the concentration used (10 µM), clotrimazole inhibited the proliferation of all tumor cell lines by 60 to 80% after five days, but did not affect the growth of PNT1A cells (Fig. 3). BCTC at 10 µM showed a similar behavior to clotrimazole, since it strongly reduced the proliferation of all cell lines (50–70%) but PNT1A. The effects of BCTC appear to occur, at least in part (see below), through its action on TRPM8, since thio-BCTC was clearly less efficacious in all lines tested. Somewhat surprisingly, DD01050 (10 µM) induced a significant inhibition only in PC3 cells to a level comparable to other inhibitors, but it did not show any significant inhibition in any other cell line (Fig. 3).

The inhibition of proliferation correlated well with the proliferative fraction which was measured as percentage of cells in S/G2/M phases of the cell cycle. As shown in Fig. 4, the relative potency of each blocker is also reflected in the decrease in proliferative fraction induced. None of the drugs tested changed the proliferative fraction of PNT1A cells. On the other hand, both clotrimazole and BCTC (both at 10 µM), decreased the cells in S/G2/M phases in all tumor cell lines, while 10 µM thio-BCTC was less potent and DD01050 was most effective on PC3 cells. We also tested the effect of AMTB and JNJ41876666, novel and highly specific TRPM8 inhibitors [37], [44], on all cell lines. At 10 µM, both drugs reduced the proliferative fraction of cancer cell lines while leaving PNT1A cells unaffected. We did not observe any cytotoxic effects of the drugs at the concentrations used (not shown). Collectively, the results obtained with the different blockers are consistent with the view that inhibition of TRPM8 results in a reduced proliferation in prostatic cancer cells.

### Knock Down of TRPM8 Message Reduces Prostate Cancer Cell Proliferation

**Figure 5:**
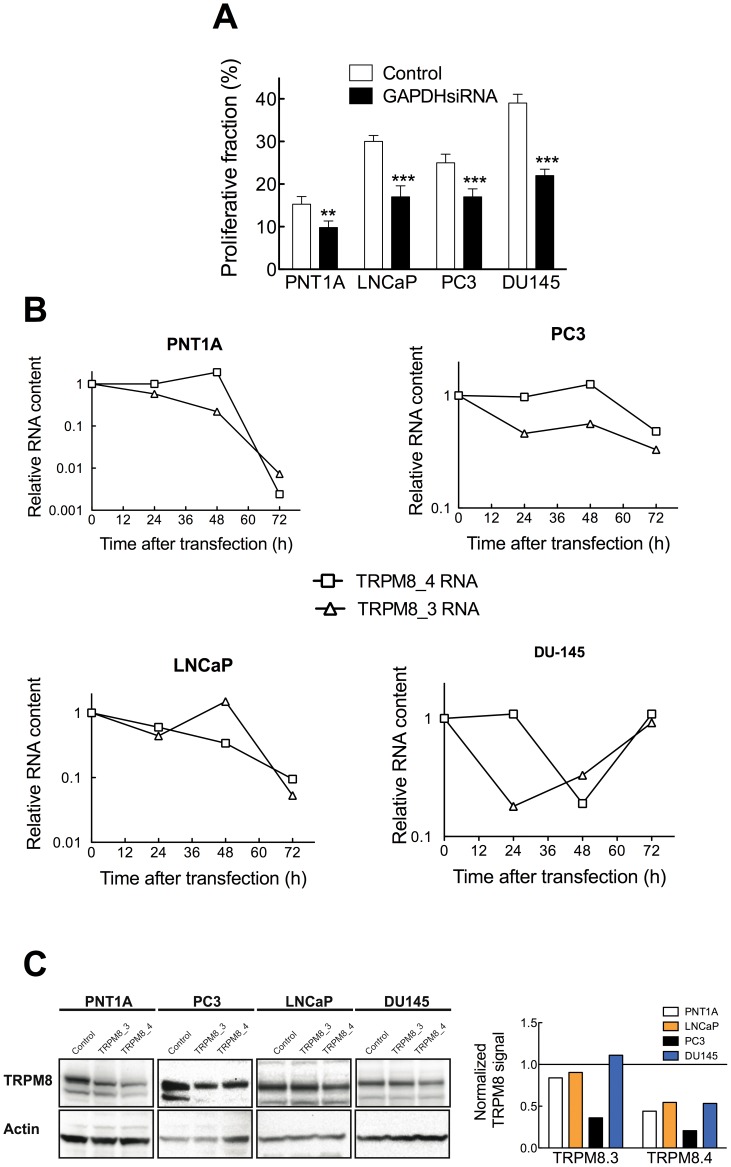
Optimization of siRNA treatment in the tested cell lines. A. The effect of GAPDH knockdown on the proliferative fraction of each cell line was determined. As expected, GAPDH knockdown inhibits proliferation in all cell types. **B.** Time course of reduction in TRPM8 RNA content by the indicated TRPM8 siRNA treatment. TRPM8 message was determined at the indicated times after transfection. Relative abundance is referred to the amount of mRNA at time 0 and corrected for the expression of transferrin receptor as control. **C.** TRPM8 protein knockdown by TRPM8.3 and TRPM8.4 siRNA 48 h after transfection. Actin was used as loading control, and the densitometric quantification relative to control siRNA is presented on the right bar diagram.

To achieve a more direct correlation between TRPM8 expression and tumor cell proliferation, we used siRNA against TRPM8 and measured its effects on proliferation and cell cycle distribution in the three tumor prostatic cell lines and the non-tumoral one. As a positive control we used siRNA directed against hGAPDH. As GAPDH is a key enzyme of glycolysis, reduction of the protein is reflected on the metabolic activity of transfected cells. This was demonstrated for the four cell lines by MTT assays (not shown) and flow cytometry (Fig. 5A).

The use of multiple functional siRNAs is advised to ensure the specificity of the phenotypic effects observed because sequences targeting different parts of the message should have qualitatively the same effects. Quantitatively, different siRNAs targeting the same gene are often not equally effective because of thermodynamic properties, stability, and positioning of either the siRNAs themselves or of the target region of the gene. We therefore tested four different siRNAs directed against TRPM8, which have been designated TRPM8.1–4. These siRNAs recognize distinct target sequences in TRPM8 and differ in their silencing potency in the different cell lines. To reduce the possibility of off-target effects, we used a low concentration of siRNA (25 nM) and optimized the transfection time. For this, we incubated each cell type with equilibrated siRNA-transfection reagent mix for 4, 6, 8, 12, and 24 h, and monitored 24 and 48 hours afterwards the relative reduction in TRPM8 elicited by TRPM8.3 and TRPM8.4 using real time PCR; TRPM8.3 and TRPM8.4 were effective at the RNA level in all four tested cell lines (including PNT1A) after incubation of 6 hours with siRNA. We also optimized the incubation time after siRNA transfection by following the abundance of TRPM8 mRNA at 12, 24, 72 and 120 hours. For PNT1A, LNCaP and PC3 the maximum reduction of TRPM8 message using TRPM8.4 siRNA was observed at 72 hours, while DU145 showed the maximum effect already at 48 hours (Fig. 5B). Silencing of TRPM8 induced a decrease in proliferation as measured by MTT assays in the PC3 cell line. MTT hydrolysis in PNT1A, DU145 and LNCaP cells was not obviously affected by siRNA (not shown). This is probably a reflection of the different optimal times for RNA knockdown, because one should expect a shift by several hours between the reduction in TRPM8 message and effects on proliferation, which will depend on the doubling time and the time needed for protein knockdown both of which are different for each cell type. The reduction of mRNA correlated with a clear reduction of protein by approximately 50% in all cell lines at 48 hours (Figure 5C), but the incomplete knockdown could explain the lack of effects on MTT hydrolysis.

**Figure 6:**
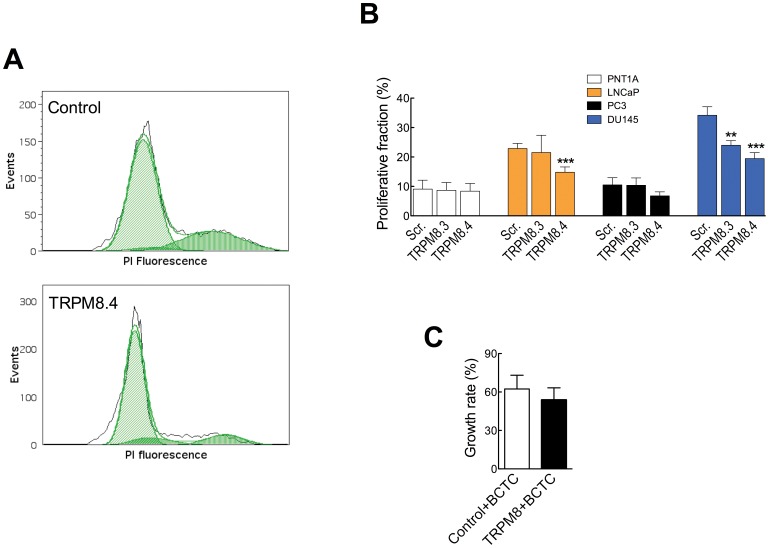
siRNA reduces the proliferative fraction of prostatic cancer cells. A . Representative cell cycle histogram of DU145 cells transfected with control (top) or hTRPM8 siRNA (bottom). The proliferative fraction (S/G2/M) is clearly reduced after TRPM8 knockdown. **B.** Proliferative fraction (%) of cells transfected with control or anti TRPM8 siRNA (sequences 3 and 4). The bars represent average of 3–5 experiments. **C.** BCTC maintains its inhibitory effects after silencing of TRPM8. Normalized growth of DU145 cells in the presence of 10 µM BCTC, after transfection of the cells with control (white column) or TRPM8.4 siRNA (black column). Growth in the absence of BCTC represents 100%.

For this reason, we performed measurements of proliferative fraction, which turned more sensitive. We detected a decrease in the fraction of cells in S/G2/M phases of the cell cycle in all tumor cell lines but observed no effect on non-tumor PNT1A cells. An example of cell cycle distribution measurements in DU145 cells is shown in Fig. 6A. A decrease in S and G2/M cells is clearly seen after siRNA treatment. The outcome of the two effective siRNAs was different depending on the cell lines; PC3 and LNCaP responded only to one of the two sequences (TRPM8.4, Fig. 6B) while for DU145 both sequences were effective.

The effects observed under siRNA made it possible to test whether the inhibition of proliferation induced by pharmacological agents is mediated solely by their effects on TRPM8. To test this, we assessed whether BCTC was able to reduce the growth of DU145 cells after siRNA treatment. If all effects of BCTC were mediated through TRPM8, the drug should have less effect on proliferation after TRPM8 knockdown by siRNA. However, BCTC (10 µM) inhibited the growth of this cell line to a similar extent after TRPM8 knockdown, indicating that the effect of BCTC on prostate cancer cell proliferation is not entirely due to TRPM8 inhibition (Fig. 6C).

Previous reports had shown that the viability of LNCaP cells is reduced by inhibition of TRPM8 [19]. We could confirm this observation, but LNCaP was the only cell line that showed this behavior. The viability for PNT1A1 was also decreased after siRNA transfection, but this effect did not depend on TRPM8, because it occurred in this cell line also for control siRNAs (data not shown).

### Menthol and Icilin Effects in Proliferation and Viability of Prostate Cells

**Figure 7:**
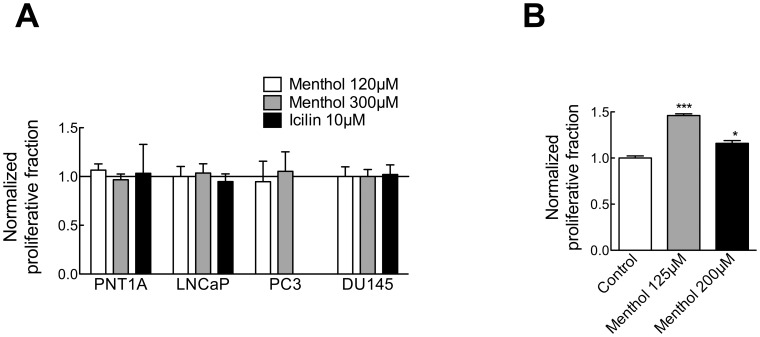
Menthol increases metabolic activity of DU145 cells but does not influence cell cycle distribution. A. The effect of menthol (two different concentrations) and icilin (10 µM) on the proliferative fraction of each cell line was determined. No effect was detected. Data are presented normalized to the proliferative fraction in the absence of TRPM8 activators. **B.** In serum-deprived cells, moderate concentrations of menthol elicited an increase in metabolic activity of DU145 cells measured by MTT assay. The effect was weaker at higher concentrations.

Our results confirm the notion that TRPM8 inhibition reduces cell proliferation of prostate cancer cells. Thus, we decided to test whether activation of TRPM8 with menthol produced an opposite effect, that is, an increase in proliferation. Menthol (120 or 300 µM) or icilin (10 µM) were added to the growth medium. Somewhat paradoxically, menthol has been described to reduce the viability of LNCaP cells [19]. The original interpretation of this phenomenon was that the activation of TRPM8 leads to a sustained Ca^2+^ influx that induces apoptosis, but menthol might have off-target effects in these cells as well [22], [45]. We did not observe a systematic reduction in LNCaP cell viability in the presence of menthol, although there was some increased mortality in some experiments. Menthol did not alter the viability of other cell lines (not shown). MTT and flow cytometry experiments in the four cell lines provided intriguing results.We did not detect any significant increase induced by menthol or icilin under normal serum concentration (Fig. 7A). However, in the presence of reduced serum, only DU145 cells showed a modest yet significant increase in proliferation induced by menthol (Fig. 7B).

### Effect of TRPM8 Block on Wound Healing

**Figure 8:**
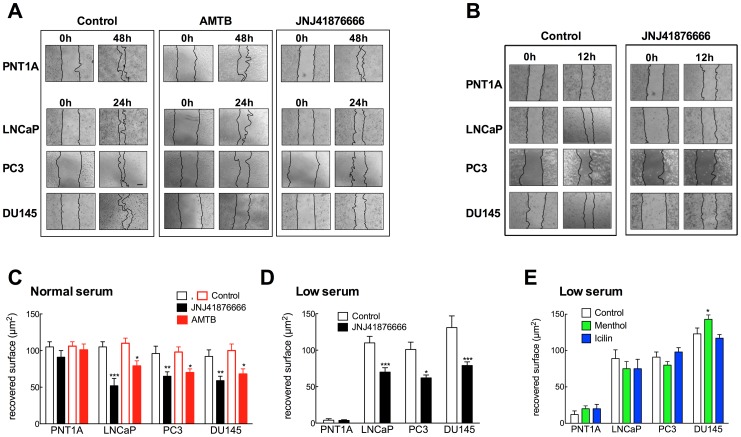
TRPM8 inhibition impairs migration of prostate tumor cells. Wound healing assays were performed on PNT1A, LNCaP, PC3 and DU145 cells in the absence or the presence of the TRPM8 inhibitors AMTB and JNJ41876666 (10 µM). A. Representative images of experiments performed in the presence of normal serum concentrations. In the absence of blocker, all cell lines efficiently restore the monolayer after 24 (LNCaP and DU145) or 48 h (PNT1A; at 24 h, no recovery was observed). The presence of blockers impairs healing in LNCaP, PC3 and DU145, but not in PNT1A cells. B. Similar effects were observed on all cell lines already 12 h after the scratch. C. Quantification of restored surface from three images like those in A, for 24 h in all cell lines, except PNT1A (48 h) in the presence of JNJ41876666 (black) and AMTB (red). D. Similar experiments in low serum concentrations revealed that PNT1A did not reduce the scratch surface, while cancer cells LNCaP and PC3 (in 3% FCS) and DU145 (in 1% FCS) did. Healing was again inhibited by JNJ41876666 (10 µM). E. Menthol induced a slight acceleration of healing only in DU145 cells, while icilin showed no effect on any of the lines tested.

Recent evidence indicates that TRPM8 activity is a relevant factor controlling migration of prostate cancer cells [24], [46]. We therefore decided to use a wound-healing assay, a classic method relaying on the combined effects of proliferation and migration [47], to study the actions of TRPM8 blockers on disrupted monolayers of LNCaP, PC3, DU145 and PNT1A cells. In the presence of normal serum concentration, all cell lines efficiently reduced the wound area; the tumor cell lines required shorter times to close the wound compared to the non-tumoral lines (Fig. 8A left, C). Wound healing was significantly inhibited by the specific TRPM8 blockers AMTB and JNJ41876666, in PC3, LNCaP and DU145 cells, but not in PNT1A (Figure 8A, C). To minimize the effects of proliferation, we performed the same analysis in all cell lines at 12 h; JNJ41876666 reduced wound healing in all tumor cell lines, but not in PNT1A cells (Fig. 8B).

In low serum concentrations, PNT1A cells failed to close the wound in 48 hours while all three cancer cell lines reduced significantly the wound surface within 24 h; under such conditions, JNJ41876666 significantly delayed wound healing in all cancer cell lines, while not in PNT1A (Figure 8D); AMTB produced essentially the same effects (data not shown). These results reinforce the idea that the effects of TRPM8 are context-dependent, and that the channel is required for wound healing in the cancer cell lines and not in the normal prostate epithelial line. We also performed scratch assays in the presence of TRPM8 activators, menthol and icilin. We observed only a modest (although significant) increase in wound healing induced by menthol in DU145 cells, while LNCaP, PC3 and PNT1A cells showed no effect (Fig. 8D). Again, these results indicate that although TRPM8 is important to sustain proliferation and migration of prostate cancer cells (but not of normal epithelium), it is not the only determinant of this process.

## Discussion

TRPM8 channels have been attributed a role in the generation and progression of prostate cancer. They were initially thought to be expressed in prostate cancer and in non-prostatic tumors (breast, colon, lung and skin) while its expression in the normal tissue was apparently almost undetectable [8], although this concept has been challenged by many other reports (e.g. [21]) and the results described here. A growing body of evidence supports the idea that the expression of TRPM8 in prostate cancer can be used as a prognostic marker and a tool for the design of novel cancer therapies [8], [19], [21], [25], [48], [49]. Furthermore, TRPM8 has even been proposed as a potential target for tumor immunotherapy [50]–[52]. Despite the growing literature regarding the physiological role of TRPM8, its role in the oncogenesis of prostate cancer remains poorly understood.

Our data are consistent with the notion that TRPM8 plays a relevant role in prostate cancer progression. Furthermore, we detected limited quantitative differences in TRPM8 expression between the tumor cell lines tested (DU145∼LNCaP>PC3), despite their radically different behavior in terms of aggressiveness, proliferation rates and differentiation. DU145, LNCaP and PC3 cells expressed both TRPM8 mRNA and protein. Furthermore, all tested cells, except PNT1A, showed a Ca^2+^ increase in response to cold that could be blocked by TRPM8 blockers [38]. The differences between our study and data of other laboratories could be due to the technique used (PCR against real-time PCR) because the PNT1A is derived from normal epithelial cells and it has been shown that there is a moderate mRNA expression in this tissue [8], [53].

At first view, the fact that TRPM8 expression is not exclusive of prostate cancer cells might be regarded as a drawback for the design of therapies based on TRPM8 inhibition. However, our pharmacological blockade and siRNA experiments strongly support the idea that the inhibition of the channel reduces the proliferation of cancer cells without affecting non-tumor cell proliferation, despite the fact that non-tumor cells also express TRPM8. In other words, TRPM8 appears to be required only for tumor cell proliferation and not for normal proliferation. Interestingly, DU145 cells, which serve as a model for aggressive androgen-independent prostate cancer, were the most sensitive cell type to proliferation inhibition by TRPM8 block. This cell line was also the only one were we observed indications that activation of the channel can enhance proliferation.

The siRNA treatment produced different effects on proliferation depending on whether the measurement was performed using MTT of by direct determination of the proliferative fraction. siRNA unequivocally reduced the proliferative fraction in all tumor cell lines, while the effects on proliferation measured as MTT hydrolysis were marginal if at all significant, except for the case of PC3 cells, which showed a dramatic decrease in MTT hydrolysis. We think that the time course of knockdown of the message, which is different for each of the cell lines, is crucial in this context. In the case of DU145 cells, the RNA level decreases very fast, but also recovers rapidly. And while PC3 cells show a steady and early-onset of RNA level reduction, it decreases only very late in LNCaP cells. This observation highlights the importance of optimization of knockdown times if indirect assays such as metabolic activity determinations are to be performed. Reduction of the message in LNCaP cells required 72 hours, comparable to their doubling time (about 60 hours). This would preclude the use of standard growth curves as a measure of the impact of siRNA treatment on proliferation. Measurement of the proliferative fraction of cells seems to be a much more reliable approach.

Additionally, our data shows that the effects of pharmacological blockers (BCTC) and siRNA are additive, indicating independent ways of action. This observation is not entirely surprising given the fact that the drugs have been described as specific in the sense that they block other channels with different efficiency. In the future, it would be desirable to repeat these experiments with more specific blockers of TRPM8 channels.

Our data confirm most of the previously reported observations in LNCaP cells [18], [19]. However, some of the new findings require alternative explanations to the prevailing view so far. For example, the described proapoptotic effect of menthol in LNCaP cells does not appear to correlate with decreased growth or loss of viability when cell cycle distribution and PI exclusion are measured. We did not observe changes in cell viability or proliferative fraction in the presence of up to 300 µM menthol or 10 µM icilin in any cell line. In as much as menthol increased [Ca^2+^] in PC3 and DU145 cells similarly to LNCaP, it is unlikely that a sustained Ca^2+^ influx is responsible for the toxic effect described for LNCaP cells. The modest effects observed could be a reflection of the low percentage of cells responding to chemical stimuli [38]. DU145 shows even increased proliferation in the presence of menthol under low serum. This effect did not occur in the presence of normal serum concentration, indicating that TRPM8 expression does not represent an advantage for growth under optimal conditions, but does improve it under limited growth factor supply. Since DU145 cells do not express androgen receptor, and the expression of TRPM8 in other cell lines has been reported to be enhanced by androgens [18] it is reasonable to speculate that DU145 is the only cell line that shows stimulated growth in the presence of menthol because it has enough expression of the channel in the absence of androgens.

Several of our observations point to a context-dependent role of TRPM8, which seems necessary for cell cycle progression and migration of LNCaP and DU145 cancer cells, while it has only small (if any) effects in non-cancer PNT1A cells and has been reported to inhibit migration of PC3 cells [24], [46]. Although this is not an unusual observation, since prominent cancer-related factors such as the TNF-related apoptosis-inducing ligand, (TRAIL e.g. [54]), retinoids [55] or IL-24 [56] can show a similar behavior, it highlights the need to carefully select appropriate models when trying to exploit the therapeutic potential of TRPM8.

In summary, we provide evidence supporting a tumor-specific *role* of TRPM8 rather than a tumor-specific *expression* of the channel, thus reinforcing the relevance of this channel as a promising candidate for prostate cancer therapy.
